# Endoscopic Endonasal Approach for Craniopharyngiomas With Intraventricular Extension: Anatomic-Clinical Considerations and Surgical Outcomes in a Series of 61 Patients

**DOI:** 10.1227/ons.0000000000001687

**Published:** 2025-08-08

**Authors:** Ilaria Bove, Domenico Solari, Teresa Somma, Riccardo Nocini, Carmela Palmiero, Felice Esposito, Paolo Cappabianca, Luigi Maria Cavallo

**Affiliations:** * Division of Neurosurgery, Department of Neurosciences, Reproductive and Odontostomatological Sciences, Universita' degli Studi di Napoli Federico II, Naples, Italy;; ‡ Unit of Otolaryngology, Head and Neck Department, University of Verona, Verona, Italy

**Keywords:** Endoscopic endonasal approach, Craniopharyngioma, Third ventricle, Transtuberculum/transplanum endoscopic endonasal approach, Hypothalamus

## Abstract

**BACKGROUND AND OBJECTIVES:**

Craniopharyngiomas (CPs) with intraventricular extension has required often a difficult surgical management. These injuries involve a high degree of endocrinological, visual, and neuropsychological morbidities, which have a huge impact on the patient's quality of life. The advancements of visualization instruments along with development of minimally invasive techniques as the endoscopic endonasal have granted reduction of morbidity and mortality rates. The aim of this retrospective study was to report our experience with the endoscopic endonasal approach in the management of a series of patients affected by CPs with intraventricular extension.

**METHODS:**

The authors reviewed data of 61 cases from a series of 164 patients, who underwent an endoscopic endonasal transtuberculum/transplanum approach for the removal of a CP involving the third ventricle between January 2001 and March 2023. Four main third ventricular growth patterns were identified: stalk-infundibulum, infundibulum-ventricular chamber, stalk-infundibulum-ventricular chamber, and ventricular chamber.

**RESULTS:**

Sixty-one patients (34 men, 27 women), with mean age of 51.87 years (range 10-79 years ± 13.66 SD), underwent extended endoscopic endonasal approach. Gross total resection was obtained in 65.6% of cases and resulted significantly influenced by the sex (95% CI, 0.080-0.60; *P* = .02), previous treatment (95% CI, 0.08-0.31; *P* = .04), and tumor location (95% CI, 0.44-0.10; *P* = .05). Postoperatively, visual improvement was observed in 40 patients (76.9%). The hypothalamic functions were improved in 6 cases (20%), remained stable in 9 (45%), instead a new-onset of hypothalamic functions disturbances were registered in 5 cases on 31 patients (16.1%). Six postoperative cerebrospinal fluid leaks (9.8%) occurred.

**CONCLUSION:**

The different topographies of intraventricular CPs affect the outcomes of resection. However, the extension of CP in the third ventricle does not represent a limit of the endonasal route; the good outcomes and limited complications confirm that.

**ABBREVIATIONS:**
CP: craniopharyngioma
DI: diabetes insipidus
EEA: endoscopic endonasal approach
EOR: extent of resection
GTR: gross total resection
NTR: near-total resection
SI: stalk-infundibulum
STR: subtotal resection.

The resection of craniopharyngiomas (CPs) involving the third ventricle can result in injury to the hypothalamus, infundibulum, pituitary gland, and optic apparatus. Maximum safe surgical resection is the primary goal according to current treatment policy, but it is often difficult to accomplish.^1-5^ Transcranial microsurgery has traditionally been the standard operative option.^6-9^ Trans-sphenoidal approaches were limited to intrasellar, infradiaphragmatic CPs. In the last decades, the evolution of transtuberculum/transplanum endoscopic endonasal approach (EEA) has given new opportunity of management for supradiaphragmatic CPs.^10-17^ According to the relationships between the tumor and the infundibulum, Kassam et al^18^ developed a classification to guide the endonasal resection of CPs. Type I to III can be surgically approached by EEA. Type IV or purely third ventricular lesions, although being amenable to EEA, should be accessed by transchoroidal or translaminar terminalis approach. Other classification schemes have been reported.^19,20^ In the “QST” type, Fan et al^21^ describe the largest surgical experience with CPs in the modern era. The authors classify CPs in 3 types according to their origin: infrasellar/subdiaphragmatic, subarachnoidal, and pars tuberalis. As previously reported by our group, we can identify 2 anatomical scenarios^10^: (1) the stalk-infundibulum (SI) complex is enlarged by the CP, and (2) the intact pituitary stalk is displaced anteriorly, medially, or laterally by a CP expanding within the third ventricle. In the first scenario, CP grows inside the infundibular recess enlarging the pituitary stalk, which together with the outer part of the infundibulum becomes a gate to enter the ventricular cavity. In the second scenario, the pituitary stalk remains a key reference landmark for defining CP's topography, and both the circumferential expansion of the infundibulum and the bulging of the third ventricle floor beneath the stalk are signs of primary tumor growth at hypothalamic level. Along with the growing experience, the EEA through 2 different corridors, ie, the suprachiasmatic through lamina terminalis or most commonly the subchiasmatic through the infundibulum, gained entering the third ventricle chamber.^10,22-24^ This study describes our experience with extended EEA for the removal of CPs involving the third ventricle, focusing on the impact of different topographies of intraventricular CPs, distinguishing between lesions involving the pituitary stalk and infundibulum (positioned lower along the hypothalamus-pituitary axis) and those occupying the infundibulum-third ventricle region (at a higher position).

## METHODS

**FIGURE 1.**
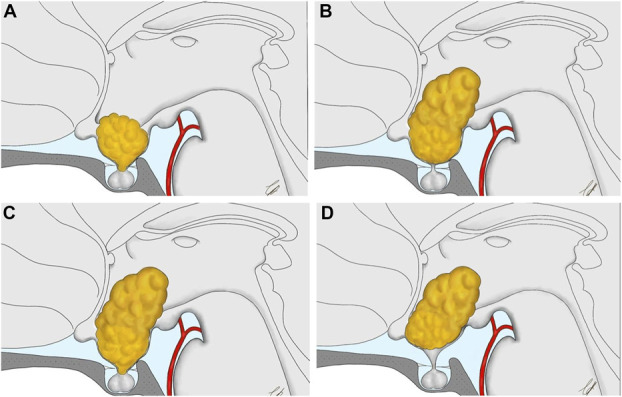
Schematic drawing showing the ventricular involvement at different level along the SI axis. **A**, SI lesion, enlarging the stalk and abutting in the infundibular region of the third ventricle. **B**, IV involvement: in this case the lesions has pushed downward the posterior wall of the infundibulum and the third ventricle floor, so that can be accessed through such path; **C**, SI-ventricular lesion in which tumor has both widening the stalk and pushed down the posterior wall of the infundibulum and the third ventricle floor; **D**, Ventricular lesion in which tumor growing within the chamber, above the level of an anatomically intact third ventricle floor. IV, infundibulum-ventricular chamber; SI, stalk-infundibulum.

Among one hundred fifty CPs undergone surgical resection by EEA at the Division of Neurosurgery between January 2001 and March 2023, we identified 61 cases (40.7%) arising or secondary extending into the ventricular chamber. The endoscopic endonasal transtuberculum/transplanum approach was performed in all cases.^10,15^ All the surgeries required dedicated team, being each procedure run according to the so-called 3 to 4 hands technique.^25^ In this scenario, 2 experienced surgeons, as best-tuned duo, have been working together. This study was conducted according to 1964 Declaration of Helsinki and its later amendments. Owing to its retrospective nature, Institutional Review Board gave its approval and waived the need for informed consent. Demographic data, preoperative assessment, tumor features, surgical results, complications, follow-up, recurrence, and hypothalamic disturbance were reported. In the study, we included both pediatric and adult patients with CP involving the third ventricle. The age of pediatric population ranged from 12 to 18 years. We selected cases with a well-pneumatized sphenoid sinus, an adequate wide chiasm-pituitary corridor, and lateral extension not beyond the optic nerve/internal carotid artery. According to our classification, we defined 4 main growth patterns of CP: SI, infundibulum-ventricular chamber, SI-ventricular chamber, and ventricular chamber (Figure 1).^10^ Extent of resection (EOR) was stated according to MRI at 3 months and classified as gross total resection (GTR) when no tumor remnant was visible, near-total resection (NTR) if ≥95% of tumor volume was resected, subtotal resection (STR) if ranging from 70% to 95%, and partial if <70%. All patients received computed tomography scan after surgery to exclude perioperative complications and thereafter underwent post-Gad MRI before being discharged to assess EOR. MRI scan at 3 months was then considered to rule out tumor remnants or regrowth that require adjuvant treatment; scans were then repeated regularly at follow-up.

### Statistical Analysis

Student *t* test or Mann-Whitney *U* test were used. For categorical variables, the difference was analyzed using the Fisher exact test. The effects of preoperative variables were analyzed using multivariate logistic regression models; in each case, *P* value < .05 was considered statistically significant. All statistical analyses were performed using R studio (Integrated Development for R. RStudio, Public-benefit Corporation).

## RESULTS

### Demographic and Clinical Data at Surgery

Among 61 patients enrolled in the study, 27 were female (44.3%) and 34 male (55.7%), and mean age was 51.87 years (range 10-79 years ± 13.66 SD). The most common presenting symptom was visual defect (73.8%), with bitemporal hemianopia in 42.3% of cases (Table 1). Rarely hydrocephalus (8.2%) and headache (6.5%) led to diagnosis of CP. Endocrinological disturbances were detected in 31 patients (50.8%): panhypopituitarism in 21.3% of cases; 9 (14.8%) presented with anterior hypopituitarism 1 axis, 2 (8.2%) presented with anterior hypopituitarism 2 axes, 2 (3.3%) with isolated diabetes insipidus (DI), and 5 (25%) with panhypopituitarism and DI. Four patients (6.5%) were admitted with manifestations consciousness impairment suggestive for intracranial hypertension. Symptoms of hypothalamic disorders were present in 30 of 61 (49.2%): obesity in most cases (39.3%). Seven patients (11.5%) experienced a previous surgical treatment; in 3 cases (3.3%), previous radiotherapy was performed. The median surgery time was 7 hours during the first 10 years; in the latter period, the median time has decreased to 5 hours. The median length of stay was 9.2 days, ranging from 7 to 30 days, depending on the clinical condition and any complications that occurred during the stay. The mean follow-up was 79.13 ± 30.74 SD months. Data are reported in Tables 1-3.

**TABLE 1.** Demographic and Clinical Data of 61 Craniopharyngiomas With Intraventricular Extension

Covariates	Overall series (61)
Demographic and clinical data	
Sex	
Female	27 (44.3%)
Male	34 (55.7%)
Age (y)	Mean 51.87 (range 10-79 ± SD 13.66)
BMI	Mean 29.2 ± 5.86 SD (range 19-49.78)
<30	37 (60.6%)
≥30	24 (39.4%)
Presenting symptoms and signs	
Preoperative visual impairment	52/61 (85.2%)
Hydrocephalus	5/61 (8.2%)
Headache	4/61 (6.5%)
Pituitary disfunction	31/61 (50.8%)
Obesity	24/61 (39.3%)
Consciousness impairment	4/61 (6.5%)
Memory disturbance	2/61 (3.2%)
Panhypopituitarism	13/61 (21.3%)
Hypopituitarism 1 axis	9/61 (14.8%)
Hypopituitarism 2 axis	2/61 (3.3%)
Panhypopituitarism + Diabetes insipidus	5/61 (8.2%)
Diabetes insipidus	2/61 (3.3%)
Bitemporal hemianopsia	22/61 (36%)
Visual acuity	20/61 (32.8%)
Bilateral quadrantanopia	3/61 (4.9%)
Unilateral quadrantanopia	2/61 (3.3%)
Unilateral hemianopsia	1/61 (1.6%)
Hemianopsia + Quadrantanopia	3/61 (4.9%)
Amaurosis + Hemianopsia/Quadrantanopia	1/61 (1.6%)
Prior surgical treatments	7/61 (11.5%)
Craniotomy	3/61 (4.9%)
Craniotomy + Ommaya	2/61 (3.3%)
EEA	2/61 (3.3%)
Radiotherapy	3/61 (3.3 %)

BMI, body mass index; EEA, endoscopic endonasal approach.

**TABLE 2.** Pathologic Data of 61 Craniopharyngiomas With Intraventricular Extension

Covariates	Overall series (61)
Tumor growth pattern	
SI	22/61 (36%)
IV	28/61 (45.9%)
SIV	9/61 (14.8%)
V	2/61 (3.3%)
Histology	
Adamantinomatous	53/61 (86.9%)
Papillary	8/61 (13.1%)
Consistency	
Mixed	44/61 (72.1%)
Solid	13/61 (21.3%)
Cystic	4/61 (6.6%)

IV, infundibulum-ventricular chamber; SI, stalk-infundibulum; SIV, stalk-infundibulum-ventricular chamber; V, purely ventricular.

**TABLE 3.** Surgical and Outcome Data of 60 Craniopharyngiomas With Intraventricular Extension

Covariates	Overall series (61)
EOR	
GTR	40 (65.6%)
NTR (70%-95%)	8 (13.1%)
STR (>70%)	13 (21.3%)
Visual outcome	
Improved	40/52 (76.9%)
Unchanged	10/52 (19.2%)
Worsened	2/52 (3.9%)
Endocrinological outcome	
Unchanged	17/31 (54.8%)
New-onset	26/30 (86.7%)
Postoperative endocrinological symptoms	
Panhypopituitarism	21/61 (34.4%)
Hypopituitarism 1 axis	17/61 (27.9%)
Hypopituitarism 2 axis	12/61 (19.7%)
Diabetes insipidus	
Unchanged	7/7 (100%)
New onset	40/54 (74.1%)
Hypothalamic disturbance	
Improved	6/30 (20%)
Unchanged	9/30 (45%)
New-onset	5/31 (16.1%)
Post BMI	
Stable	37/61 (60.7%)
<10 kg weight gain	15/61 (24.6%)
10-20 kg weight gain	6/61 (9.8%)
>20 kg weight gain	3/61 (4.9%)
Complications	
CSF leak	6/61 (9.8%)
Subdural hematoma	2/61 (3.25%)
Hemorrhage	2/61 (3.25%)
Cranial nerves palsy	1/61 (1.6%)
Meningitis	1/61 (1.6%)
Hydrocephalus	2/61 (3.25%)
Death related complication	1/61 (1.6%)
Death related disease progression	2/61 (3.25%)
Recurrence	4/61 (6.5%)
Adjuvant treatments	
Radiation therapy	21/61 (34.4%)
Immunotherapy	1/61 (1.6%)
PFS	27 mo
Follow-up	79.13 ± 30.74 SD

BMI, body mass index; CSF, cerebrospinal fluid; EOR, extent of resection; GTR, gross total resection; NTR, near total resection; PFS, progression-free survival; STR, subtotal resection.

### Tumor Features

Two cases (3.3%) were purely intraventricular CPs. Most of the lesions (96.7%) presented with various degree of secondary involvement: 22 (36%) involved the SI, 28 (45.9%) the infundibulum-ventricular chamber, and 9 (14.8%) the SI-ventricular chamber. The pathological report disclosed 53 cases (86.9%) of adamantinomatous CPs, whereas 8 (13.1%) were papillary. Tumors were defined as mixed solid-cystic (72.1%), solid (21.3%), and cystic (6.6%), respectively (Table 2).

### EOR and Surgical Outcomes

Postoperative visual function improved in 40 cases (76.9%), remained stable in 10 (19.2%), and in 2 cases (3.9%) worsened. Regarding the postoperative endocrinological status, primary condition was found unchanged in 54.8%. New-onset of postoperative pituitary gland dysfunction was reported in 86.7% (new hypopituitarism: 19, new pan hypopituitarism: 6, new DI: 5). The DI remained unchanged after surgery in every case (Table 3). An improvement in intellectual ability was observed in 6 patients (20%), no change in 9 patients (45%), and deterioration in 5 cases (16.1%). Body mass index remained unchanged in 37 patients (60.7%), whereas in 15 cases (24.6%), we observed weight gain lesser than 10 kg, in 6 (9.8%) between 10 and 20 kg, and in 3 (4.9%) more than 20 kg. We did not find a statistically significant effect of third ventricle involvement with hypothalamic disturbance (*P* > .5). GTR was achieved in 40 patients (65.6%), a NTR in 8 (13.1%), and STR in 13 (21.3%). The EOR resulted significantly influenced by the sex (95% CI, 0.080-0.60; *P* = .02), previous treatment (95% CI, 0.08-0.31; *P* = .04), and tumor location (95% CI, 0.44-0.10; *P* = .05). Female sex, previous treatment (ie, surgery or/and radiotherapy), and SI group were associated with low incidence of GTR (Table 4). The postoperative cerebrospinal fluid (CSF) leakage rate was 9.8% (5/61); it occurred in 4 of the 21 patients (19.04%), who received gasket seal/grandma cap reconstruction technique^26,27^; in 1 of 17 (5.9%) in whom multilayer sandwich technique was adopted; and in 1 of 23 patients in whom 3F reconstruction technique was performed.^28,29^ Of these patients, 5 presented with frank postoperative CSF leakage that required second surgery: in 4 cases, a watertight reconstruction was achieved using de novo gasket seal technique, whereas in 1 case, the multilayer sandwich technique was adopted. In a single case, small weeping was observed, so awake sealant technique was used to seal the CSF leakage.^30^ Lumbar drain was not used preoperatively or postoperatively in any cases, as well as no patient required lumbar-peritoneal shunts. One patient with retrosellar lesion suffered on postoperative day 1 of transient third cranial nerve palsy in the right eye (1.6%); we also observed 2 cases of subdural hematoma (3.25%, 2/61), 2 cases of hemorrhage (3.25%, 2/61), 2 cases of hydrocephalus (3.25%, 2/61), and 1 case of meningitis (1.6%, 1/61). Three patients (4.9%) died during follow-up: 2 for progression disease (3.25%, 2/61) and 1 for meningitis complication (1.6%, 1/61) (Table 5).^31^ On discharge, we recommend nasal rinsing with a topical nasal spray, usually 10 to 15 days after surgery. For up to 4 to 6 weeks, patients are advised to avoid straining and any activities that may promote Valsalva maneuvers and/or leaning the head forward.^32^ Postoperative nasal discomfort was not reported in any case.

**TABLE 4.** Tumor Features, Outcome, and Extent of Resection

Tumor features	GTR (65.6%) N = 40	NTR (13.1%) N = 8	STR (21.3%) N = 13	*P*-value
Gender female	13 (32.5%)	5 (62.5%)	9 (69.2%)	**.02**
Previous treatment	3 (7.5%)	2 (25%)	2 (15.4%)	**.04**
Age (y)	51.3	50.77	49.64	.83
Location				
SI	12 (30%)	5 (62.5%)	6 (46.1%)	**.05**
SIV	6 (15%)	0	2 (15.4%)	.4
IV	22 (55%)	3 (37.5%)	3 (23.1%)	.2
V	0	0	2 (15.4%)	.76
Consistency				
Cystic	3 (7.5%)	1 (12.5%)	0	.88
Solid	10 (25%)	1 (12.5%)	2 (15.4%)	.36
Mixed	27 (67.5%)	6 (75%)	11 (84.6%)	.3
Size (max diameter, cm)				
<3	19 (47.5%)	N = 3 (37.5%)	5 (38.4%)	.68
>3	21 (52.5%)	N = 5 (62.5%)	8 (61.5%)	
Pathology				
Adamatinomatosus	38 (95%)	N = 8 (100%)	7 (53.8%)	**.05**
Papillary	2 (5%)	0	6 (46.2%)	
CSF leak	4 (10%)	1 (12.5%)	1 (7.7%)	.3
Recurrence rate	2 (5 %)	1 (12.5%)	1 (7.7%)	.99

CSF, cerebrospinal fluid; GTR, gross total resection; IV, infundibulum-ventricular chamber; NTR, near total resection; SI, stalk-infundibulum; SIV, stalk-infundibulum-ventricular chamber; STR, subtotal resection; V, purely ventricular.

Bold represents statistically significant data (*P* < .05).

**TABLE 5.** Tumor Complication and Intraventricular Craniopharyngioma Topography According to Our Previous Classification

Tumor features	SI (36%) (N = 22)	SIV (14.8%) (N = 9)	IV (45.9%) (N = 28)	V (3.3%) (N = 2)	*P*-value
CSF leak	0	N = 1 (12.5%)	N = 5 (17.85%)	0	.32
Hemorrhage	N = 2 (8.7%)	0	N = 2 (7.1%)	0	.98
Cranial nerves palsy	0	0	0	N = 1 (50%)	.15
Meningitis	0	14.3% (N = 1)	0	0	.17
Hydrocephalus	0	N = 1 (11.1%)	0	N = 1 (50%)	**.04**
Diabetes insipidus	N = 15 (65.2%)	N = 6 (75%)	N = 21 (75%)	N = 2 (100%)	.4

CSF, cerebrospinal fluid; IV, infundibulum-ventricular chamber; SI, stalk-infundibulum; SIV, stalk-infundibulum-ventricular chamber; V, purely ventricular.

Bold represents statistically significant data (*P* < .05).

### Follow-Up

During the follow-up (mean 79.13 ± 30.74 SD months), tumor recurrence was registered in 4 cases (6.5%) with a mean progression-free survival of 27 months: 2 of them undergone GTR (1 was previously treated through EEA + Ommaya reservoir positioning and by pure EEA), 1 after NTR, and the 1 after STR. Adjuvant stereotactic radiosurgery was administered in 21 patients (34.4%), of which 10 after NTR, 7 after STR, and 4 after GTR. One patient undergone STR of a papillary CP harboring BRAF (V600E) mutation, received adjuvant treatment with BRAF/methyl ethyl ketone inhibitor and the MRI at last follow-up disclosed no tumor.^33^ In univariate analysis, risk of recurrence was not significantly increased with tumor removal (*P* = .99). No significant difference was found in the Kaplan-Meier analysis regarding progression-free survival rate according to tumor resection (*P* = .5).

## DISCUSSION

CP is a histologically benign but locally aggressive tumor, which can arise along hypothalamus-pituitary axis.^3,34,35^ The involvement of the third ventricular chamber is quite common, either due to the extension of lesions from their origin in the sellar-suprasellar area or, less frequently, from growth of the ventricular floor lining cells. There is not a univocal consensus in the literature regarding the optimal treatment strategy for CPs.^1,5,34,36,37^ Multiple classifications have been proposed to support the choice of the best management strategy on anatomical tumor features and functional considerations.^38,39^ To strengthen the results of the present series, we compared our data with those retrieved from the literature. We summarized the outcomes observed in surgical series of CPs extending into the ventricular chamber and purely third ventricle CPs. Specifically, we focused on third ventricle CPs treated by EEA or transcranial approach from January 2000 to December 2024 (Table 6).^10,39-60^

**TABLE 6.** Previous Studies Reporting Series of Transcranial Approach and EEA of Intraventricular Craniopharyngiomas in the Literature After 2000

Authors	No. of pts	Third ventricular involvement	Preoperative endocrinological status	Postoperative endocrinological disorder	Postoperative visual disorder	Approach	EOR	Other complications	Recurrence	Mean Follow-up (mo)
Chen^40^	1	ITVC	NA	New onset DI 1 (100%)	NA	TCA	GTR 100%	NA	0	24
Behari et al^41^	6	ITVC	NA	API 2 (33%), New onset DI 1 (17%)	NA	TCA	GTR 50%	Hydrocephalus 1 (17%)	0	20
Maira et al^42^	8	ITVC	NA	API 2 (25%)	NA	TCA	GTR 88%	Neurological deterioration 2 (25%)	NA	117
Steno et al^43^	29	3CVP	NA	NA	NA	TCA	GTR 75.9%	NA	7 (24.1%)	54.5
Pasqual et al^44^	1	ITVC	NA	NA	NA	TCA	NTR 100%	NA	NA	NA
Madhavan et al^45^	1	ITVC	NA	NA	NA	TCA	—	Death for myocardial infarction	NA	NA
Agrawal et al^46^	1	ITVC	NA	NA	0	TCA	GTR 100%	—	NA	NA
Tayari et al^47^	1	ITVC	NA	NA	NA	TCA	GTR 100%	—	0	NA
Pan Jun et al^48^	17	ITVC	DI 8 (61.5%) HT 6 (46.2%) API 3 (23.1%)	API 4 (30.8%) HT 5 (38.5%) DI 5 (38.5%)	NA	TCA	GTR 76.5%	Epidural hematoma with death (1/7.7%)	3 (30.8%)	NA
Jung et al^49^	4	ITVC	DI 1 (50%)	API 2 (50%)	0	TCA	GTR 100%	NA	2 (50%)	59
Yu et al^50^	24	ITVC	DI 5 (20.8%)	Partial API 8 (33.3%) Corticotroph insufficiency 13 (54.2%) New onset DI 15 (62.5%)	NA	TCA	GTR 79%	Memory loss 1 (4.1%)	6 (25%)	42
Cavallo et al^10^	12	3CVP	API + DI 2 (16.7%) API 6 (50%)	Partial API 1 (8.3%) New onset DI 6 (50%)	1 (8.3%)	EEA	NA	CSF leak 2 (16.7%) CSDH 2 (16.7%)	0	31.4
Gu et al^51^	3	3CVP	HT + HPRL 3 (100%)	0	0	EEA	GTR 100%	CSF leak 1 (33.3%)	0	35.6
Nishioka et al^52^	3	ITVC	NA	API + New onset DI 3 (100%)	0	EEA	GTR 100%	NA	0	18.3
Forbes et al^53^	10	ITVC	HG 3 (30%) HG + HT 3 (30%) DI 2 (20%)	New onset DI 5 (50%) API 9 (90%)	1 (10%)	EEA	GTR 90%	CSF leak 1 (10%) FUO 1 (10%)	2 (20%)	46.8
Cai et al^54^	27	TVC	Partial HP 16 (59.3%) PanHy 2 (7.4%) DI 5 (18.5%)	API 11 (40.1%) DI 10 (45.5%)	1 (3.7%)	TCA 63% TCA endoscope assistant 37%	GTR 85.2%	Memory loss 1 (3.7%) Pseudomeningocele 2 (7.4%) Sellar hematoma 1 (3.7%)	1 (3.7%)	49.6
Hung et al^55^	5	ITVC	NA	NA	NA	TCA	NA	NA	NA	NA
Deopujari et al^39^	25	ITVC	11 (44%)	NA	DI 7 (25%)	TCA 18 (72%) EEA 7 (28%)	GTR 40%	Meningitis 2 (8%) Hydrocephalus 4 (16%) Death 3 (12%)	5 (20%)	
Fan et al^56^	26	3CVP	Partial API 7 (26.9%) DI 1 (3.8%)	NA	NA	EEA	GTR 92.3%	NA	1 (3.8%)	24.2
Cao et al^57^	11	TVC	API 0 DI 3 (27.3%)	API 7 (63.7%) New onset DI 5 (45.5%)	2 (18.2%)	EEA	GTR 72.3%	Meningitis 1 (9%) Electrolyte imbalance 7 (63.7%)	1 (9.1%)	12.16 ± 3.40
Zoli et al^58^	36	3CVP	API 15 (41.6%) DI 2 (5.6%) API + DI 9 (25%)	Partial API 35 (97.2%)	2 (5.5%)	EEA	GTR 91.7%	CSF 5 (13.9%) Meningitis 3 (8.3%) third ventricle hematoma 1 (2.8%) Epistaxis 1 (2.8%) transitory palsy of the third cranial nerve 1 (2.8%) Transitory memory disturbance 1 (2.8%)	6 (16.7%)	43 ± 38
Cao et al^59^	22	ITVC	DI 5 (22.7%) HG 4 (18.2%) Partial API 2 (9.1%)	API 15 (68.2%)	2 (9.1%)	EEA	GTR 95.5%	CSF leak 1 (4.6%)	2 (9.1%)	22.16 ± 7.41
Zhou et al^60^	14	ITVC	Partial API 5 (35.7%) DI 1 (7.1%)	API 5 (35.7%) New onset DI 1 (7.1%)	1 (7.1%)	EEA	GTR 92.8%	NA	O	26.2
Current study	61	3CVP	API 13 (21.3%) Hypopituitarism 1 axis 9 (14.8%) Hypopituitarism 2 axis 2 (3.3%) API + DI 5 (8.2%) DI 2 (3.3%)	API 21 (34.4%) Hypopituitarism 1 axis 17 (27.9%) Hypopituitarism 2 axis 12 (19.7%) New onset DI 40 (74.1%)	2/52 (3.9%)	EEA	GTR 65.6%	CSF leak 6/61 (9.8%) Subdural Hematoma 2 (3.25%) Hemorrhage 2 (3.25%) Cranial nerves palsy 1 (1.6%) Meningitis 1 (1.6%) Hydrocephalus 2 (3.25%) Death related complication 1 (1.6%) Death related disease progression 2 (3.25%)	4/61 (6.5%)	79.13 ± 30.74 SD

3CPV, third ventricle craniopharyngioma; API, anterior pituitary insufficiency; CSDH, cerebral subdural hematoma; CSF, cerebrospinal fluid; DI, diabetes insipidus; EEA, endoscopic endonasal approach; EOR, extent of resection; FUO, unknown fever; GTR, gross total resection; HG, hypogonadism; HPRL, Hyperprolactinemia; HT, hypothyroidism; ITVC, intrinsic third ventricle craniopharyngioma; NA, not applicable; NTR, near total resection; TCA, transcranial approach.

### Surgical Consideration

CP, growing within the infundibular recess, typically expands the pituitary stalk, which, along with the infundibulum, serves as a gateway into the ventricular cavity. Tumor can move anteriorly the optic chiasm in a position of “prefixed chiasm”; in these cases, patients tend to have a narrow chiasm-pituitary corridor, whereas postfixed chiasms have a large chiasm-pituitary corridor.^61,62^ Our data reveal that neither the location of the chiasm nor the size of the corridor between the top of the pituitary gland and the bottom of the chiasm should be considered an absolute contraindication for CP resection, thanks to gateway of the infundibulum. To avoid damage to the floor of the third ventricle, tumor removal begins with the superior intraventricular component, followed by the inferior portion along the floor of the third ventricle. This approach allows for better visualization of the hypothalamus and the arterial perforator vessels of the pituitary stalk and optic chiasm.

Preoperative MRI key factors:

1. Chiasm position: Prefixed position of the chiasm requires extreme care during tuberculum sellae bone opening, but, on the other hand, it allows an easier way to get into the subchiasmatic space;
2. Chiasm-pituitary gland distance: A narrow chiasm-pituitary corridor can be considerate a relative contraindication of EEA;
3. Position of the third ventricle floor: The floor of the 3D ventricle can be infiltrated at the level of tuber cinereum or at the infundibular recess, and the tumor can extend outside the ventricle in the interpeduncular cistern;
4. Mammillary bodies–dorsum sellae distance: This distance is crucial for tumor dissection from the floor of the third ventricle, as the mammillary bodies mark the anterior limit of the brainstem;^19^
5. Sphenoid sinus: A well-pneumatized sphenoid sinus allows a better visualization of sellar anatomic landmarks. Nevertheless, none of the anatomic variations of sphenoid sinus can be considered an absolute contraindication.

The intraventricular component, compressing the lateral wall of the third ventricle from within, leads to widening of its floor. This, in turn, facilitates the infrachiasmatic corridor and simplifies tumor dissection maneuvers.^10,23^ The GTR was obtained in most of the cases (65.6%) and resulted significantly influenced by the sex (*P* = .02), previous treatment (*P* = .04), and tumor location (*P* = .05). SI involvement was associated with low incidence of GTR. This variability depends on the growth pattern itself and the maneuvers adapted to preserve neurovascular structures, especially at the infundibulum where the tumor does not enlarge enough it. Adhesions and scar tissue in the surgical field from previous treatments affect the EOR. Regardless of the surgical approach used, the main problem of recurrent CP surgery is the loss of gliotic reaction between the tumor and the surrounding neural tissue, further complicated by the presence of arachnoid scars. As previously reported by our group, the use of the expanded endoscopic approach for recurrent and residual CPs improves the identification of boundaries between tumor and normal tissue, allowing a more secure and radical excision.^63^ Concerning the hypothalamic-pituitary dysfunction, several factors should be considered, ie, age of onset, histological subtype, tumor location, and recurrence. Guo et al^64^ found that hypothalamic syndrome was related to older age of onset, tumor recurrence, adamantinomatous type, and high preoperative neuroendocrine dysfunction. Previous studies have shown that weight gain may be related to suprasellar and invasive tumor behavior, which led damage of the paraventricular and the suprachiasmatic nucleus.^65^ In a multidimensional analysis of brain structure/function by Lee et al.^66^, psychological and behavioral phenotypes showed that patients with hypothalamic disorders have lower neural activation in the left caudate nucleus in response to food imagery. Hypothalamic involvement has been reported to have a statistically significant negative effect on overall 20-year survival in children with CP, so understanding the pathological mechanisms could open new avenues for targeted psychobehavioral therapeutics.^67^ It is worth noting that after extensive manipulation of the nasal mucosa, postoperative nasal discomfort including nasal crusting, discharge, and obstruction, with transient hyposmia, might occur. Although we did not conduct any quality-of-life assessment, through questionnaire or otolaryngology direct observation, we can say that major nasal discomfort was avoided, thanks to our strategy. Indeed, along years, with increasing confidence, we adopted a “nasal-sparing” technique; hence, we prioritized meticulous postoperative care, including the use of topical nasal sprays, which resulted in complete restoration of nasal function. The major drawback of this route is the higher risk of CSF leak.^38,53^ No statistical difference was found regarding CSF leak and CPs with intraventricular extension topography (*P* = .3); contrariwise difference was found for postoperative hydrocephalus (Table 5). Nevertheless, the introduction of nasoseptal flap to bolster the reconstruction that our school adopted in the so-called 3F technique reduced the risk of CSF leak.^29-68^ In the 3F technique, fat pad is used as a cork through the osteodural break, then the flap is raised and reflected over the skull base defect, and the patient is mobilized as fast as possible.^29^ The postoperative CSF leak in our series dropped significantly from 13.2% (5/38) using multilayer reconstruction technique—ie, autologous, heterologous, and/or synthetic, materials used in different methods—to 0 cases on 23 after the introduction of 3F technique.

### Limitations

The mechanisms underlying hypothalamic disturbance are multifactorial; in our study, we evaluated only some aspects inherent in the eating behavior, sleep-wake quality, and memory disturbance through patient communication. Future studies and the use of specific psychobehavioral scales will help us better understand the biological mechanisms and neural target involved in patients with intraventricular CP.

## CONCLUSIONS

Our study evaluates the possible impact of different topographies of intraventricular CPs on clinical outcomes distinguishing between lesions involving the pituitary stalk and infundibulum and tumor occupying the infundibulum-third ventricle region. Our results demonstrate that SI involvement was associated with lower incidence of GTR; this variability depends on maneuvers adapted to preserve neurovascular structures, especially at the infundibulum where the tumor does not enlarge it. Our data enforce the role of EEA as a safe and effective surgery for radical resection and low incidence of hypothalamic-pituitary complications for intraventricular CPs. Nonetheless, it is essential to refine surgical strategy that takes into account the anatomy and key features of the region.
